# Iron Metabolism and Ferroptosis in Epilepsy

**DOI:** 10.3389/fnins.2020.601193

**Published:** 2020-12-08

**Authors:** Shuang Chen, Yongmin Chen, Yukang Zhang, Xi Kuang, Yan Liu, Meiwen Guo, Lin Ma, Daqi Zhang, Qifu Li

**Affiliations:** ^1^Department of Neurology, The First Affiliated Hospital of Hainan Medical University, Haikou, China; ^2^Key Laboratory of Brain Science Research and Transformation in Tropical Environment of Hainan Province, Hainan Medical University, Haikou, China; ^3^Hainan Health Vocational College, Haikou, China; ^4^Department of Rehabilitation, Hainan Cancer Hospital, Haikou, China

**Keywords:** epilepsy, iron metabolism, ferroptosis, GPX4, cell death, post-traumatic epilepsy, autophagy

## Abstract

Epilepsy is a disease characterized by recurrent, episodic, and transient central nervous system (CNS) dysfunction resulting from an excessive synchronous discharge of brain neurons. It is characterized by diverse etiology, complex pathogenesis, and difficult treatment. In addition, most epileptic patients exhibit social cognitive impairment and psychological impairment. Iron is an essential trace element for human growth and development and is also involved in a variety of redox reactions in organisms. However, abnormal iron metabolism is associated with several neurological disorders, including hemorrhagic post-stroke epilepsy and post-traumatic epilepsy (PTE). Moreover, ferroptosis is also considered a new form of regulation of cell death, which is attributed to severe lipid peroxidation caused by the production of reactive oxygen species (ROS) and iron overload found in various neurological diseases, including epilepsy. Therefore, this review summarizes the study on iron metabolism and ferroptosis in epilepsy, in order to elucidate the correlation between iron and epilepsy. It also provides a novel method for the treatment, prevention, and research of epilepsy, to control epileptic seizures and reduce nerve injury after the epileptic seizure.

## Introduction

Epilepsy is a disease characterized by recurrent, episodic, and transient CNS dysfunction resulting from an excessive synchronous discharge of brain neurons and both the morbidity and mortality are high. Epileptic seizures lead to cognitive impairment in patients with respect to execution ability, language ability, attention, judgment, and mental disorders, thereby severely affecting the employment ability and social communication level of epilepsy patients and reducing the quality of life (Fazel et al., 2013; Witt and Helmstaedter, 2015; Leeman-Markowski and Schachter, 2016). Currently, AEDs are the primary methods of treatment for epilepsy. Moreover, the drug-refractory epilepsy patients accounted for about 1/3rd of the total epilepsy patients who showed no apparent response to the commonly used AEDs (Moshé et al., 2015). The occurrence of epilepsy is usually a self-facilitated pathological process triggered by the initial brain damage, ultimately leading to neurological damage, ionic pathway dysfunction, mossin fibrosis, and glial proliferation synaptic plasticity, and inflammatory response and affecting the nerve function of the brain (Gan et al., 2015). Although the abnormal discharge of neurons is a leading pathophysiological manifestation of epilepsy, owing to its complex and changeable property, the mechanism of epilepsy is yet unclear, which renders difficulty in the prevention and treatment of epilepsy.

Iron is an essential trace element for the growth and development of humans. Iron compounds are also involved in many redox reactions: oxygen transport, cellular oxidative respiratory chain, tricarboxylic acid cycle, and DNA biosynthesis (Thirupathi and Chang, 2019; Abbina et al., 2020). However, in the nervous system, iron is closely related to the formation of myelin, the metabolism of catecholamine neurotransmitters, and is also involved in intellectual development and neurodegenerative diseases (Thirupathi and Chang, 2019). Therefore, in the human body, iron metabolism should be strictly regulated. Abnormal iron metabolism is associated with a variety of neurological diseases, including hemorrhagic post-stroke and PTE (Yokoi et al., 1995; Mori et al., 2004). Ferroptosis is a new form of regulated cell death, attributed to severe lipid peroxidation caused by the production of ROS and iron overload and detected in several neurological diseases, including epilepsy (Kahn-Kirby et al., 2019). Ferroptosis is different from apoptosis, necrosis, and autophagy in terms of morphology and biochemistry. Some studies have shown that ferroptosis regulates nerve cell death in a variety of neurological diseases, including epilepsy (Kahn-Kirby et al., 2019; Chen J. et al., 2020). Therefore, understanding the regulatory mechanisms of brain iron metabolism and ferroptosis in epilepsy is essential. Thus, discovering new therapeutic targets related to iron metabolism and ferroptosis and exploring the correlation between brain iron and the occurrence of epilepsy are crucial for the prevention and treatment of epilepsy. This review summarizes and discusses the treatment of epilepsy by iron metabolism and ferroptosis and provides a new direction for the study of pathogenesis, treatment, and prevention of epilepsy.

## Iron-Induced Epilepsy

Currently, iron overload is one of the common causes of refractory epilepsy in patients with hemorrhagic stroke. When a brain injury or hemorrhagic cortical infarct occurs, it leads to blood extravasation and the damage of erythrocytes and hemoglobin. Hemoglobin and iron released by hemoglobin are associated with ROS and RNS (Mori et al., 2004). However, ROS and RNS have been shown to be associated with iron-induced epileptic seizures in rats. In epilepsy, PTE is one of the most common and devastating complications of TBI (Glushakov et al., 2016). Briefly, TBI results in the occurrence and development of epilepsy, which might be related to the breakdown of red blood cells and hemoglobin in the CNS. Current studies have shown that injections of hemoglobin or iron salts (ferric chloride, FeCl_3_) into the cortex of rats can cause chronic epileptic seizures. The O^2–^ are formed, and OH. are produced after the injection of FeCl_3_ into the cerebral cortex of rats. These free radicals might cause lipid peroxidation in neuronal membranes and the accelerated production of guanidine compounds in the brain that leads to epilepsy (Mori et al., 1990). Also, hemolysis and hemoglobin deposition occur in the neocortex after hemorrhage in the villus blood vessels. Next, iron from the breakdown and transfer of hemoglobin is deposited in the brains of individuals with PTE, which can form reactive free radical oxidants (Willmore and Ueda, 2009). Microinjections of trivalent iron ions in rodent brain cause chronic recurrent seizures and release glutamate into nerve fibers, as observed in epileptic patients (Willmore and Ueda, 2009).

In the current study of epilepsy, a chronic recurrent seizure was induced by injecting FeCl_3_ into the cerebral cortex of rats, thereby simulating hemorrhagic post-stroke epilepsy and PTE (Yokoi et al., 1995). Furthermore, the injection of nanoscale iron into the cortex induces chronic epilepsy in mice and simulates brain injury due to micro-hemorrhage, which in turn, gives rise to different degrees of spontaneous epileptiform events (Mori et al., 1990). In addition, the severity of epileptiform events is associated with the reduction of γ-aminobutyric acid (GABA) neurons in the iron-injected hemisphere and the degree of cerebral blood flow autoregulation dysfunction (Jyoti et al., 2009). In conclusion, the epileptic seizures can be induced by injecting FeCl_3_ into the cerebral cortex, thereby simulating hemorrhagic post-stroke epilepsy and PTE. This induction of epilepsy suggests that iron plays a major role in the occurrence of epilepsy and contributes to the mechanism and treatment of hemorrhagic post-stroke epilepsy and PTE.

## Iron Metabolism in Epilepsy

In the brain, the degradation of hemoglobin leads to the deposition of hemosiderin, which is one of the main forms of stored iron in the human body and closely related to neurological disorders, such as epilepsy (Zhang L. et al., 2018). The iron in the human body is absorbed as divalent iron and oxidized into trivalent iron by ceruloplasmin in the blood (Mukhopadhyay, 2018; Naito et al., 2020). The cytokines (IL-6 and TNF-α) are associated with iron regulation and metabolism (Tombini et al., 2013), while epilepsy is closely associated with inflammation (Sanz and Garcia-Gimeno, 2020). This phenomenon suggested that iron influences the occurrence and development of epilepsy by regulating inflammation in the epileptic brain. To test the hypothesis that iron overload leads to epilepsy, transferrin saturation was measured in 130 epileptic patients. The results showed that the average transferrin saturation of epileptic patients was significantly higher than that of the control group (Ikeda, 2001). The changes in the whole-brain iron in MTLE were studied by sensitivity-weighted imaging (SWI). The findings revealed that iron in MTLE Is redistributed between subcortical and cortical structures, and the degree of redistribution is influenced by the progression of epilepsy and precipitation factors (Zhang et al., 2014). The study of cerebral iron redistribution provides new insights into the pathogenesis of MTLE, deeming it as a potential biomarker for monitoring the clinical progression of epilepsy. However, some studies have shown inadequate levels of copper and manganese in the hippocampus in patients with MTLE and hippocampus sclerosis (MTLS-HS), while iron levels remain unaltered (Aleksandar et al., 2014). It was found that during the absorption process, citrate enhances the uptake of iron, which in turn, promotes the absorption of citrate. The iron-citrate uptake was coupled to sodium (Na) in the neuronal cell. The Na-coupled iron-citrate transport system can deliver citrate into the affected neurons independent of the Na-coupled transporter and may provide a novel treatment strategy for epilepsy. This phenomenon also suggested that iron overload can stimulate cells to absorb citrate, thereby affecting metabolism (Ogura et al., 2018).

In summary, abnormal iron metabolism and distribution in the brain of epileptic patients might be one of the reasons for the occurrence and development of epilepsy. Although the injection of iron into the brain induce epilepsy, the correlation between epilepsy and iron metabolism is not yet clarified and needs further exploration. We can also use imaging methods to diagnose and explore epilepsy through the abnormal distribution of iron in the epileptic brain.

## Ferroptosis and Epilepsy

### Mechanisms of Ferroptosis

**FIGURE 1:**
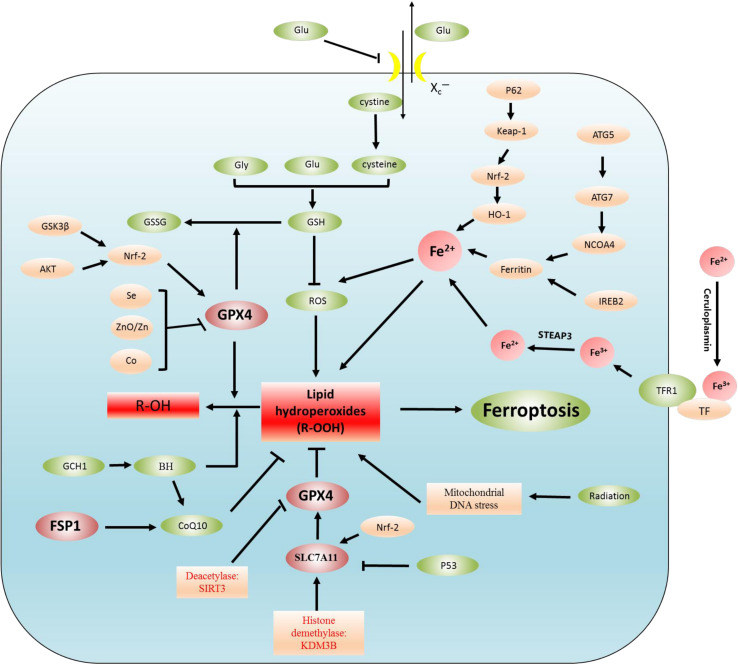
Regulatory mechanisms of ferroptosis. Lipid peroxidation leads to ferroptosis. The regulation of lipid peroxidation is related to the following regulation pathways. (1) System Xc^–^-CSH-GPX4 pathway, P53-SLC7A11-GPX4 pathway, (2) Akt-Nrf2-GPX4 pathway, GSK3β-NRF2-GPX4 pathway, Se/Zn/Co-GPX4 pathway, (3) Autophagy-dependent ferroptosis: NCOA4-induced ferritinophagy (ferritin is degraded by autophagy), (4) Iron metabolism: p62-Keap1-NRF2-Fe^2+^ regulatory pathway, IREB2-ferritin-Fe^2+^ regulatory pathway. In addition, Fe^3+^ binds to plasma TF to transport iron to all organs, TF delivers iron into cells by binding to TFR1, and Fe^3+^ is released from TF. Fe^3+^ is reduced to Fe^2+^ by STEAP3, (5) FSP1-CoQ10 pathway: the FSP1-CoQ10 pathway directly regulates lipid peroxidation independent of GPX4.

Ferroptosis is the result of the simultaneous action of multiple biological pathways. It is also considered a new form of cell death regulation, attributed to severe lipid peroxidation caused by ROS and iron overload and found in a variety of neurological diseases, including epilepsy. Importantly, ferroptosis is related to ROS, redox glutathione (GSH), GPX4, iron ions (Fe^3+^, Fe^2+^) in cells, and lipid peroxidation, which involves some pathways (Figure 1 and Table 1). In addition, we also summarized the inhibitors of ROS, GSH, GPX4, iron ions, and lipid peroxidation (Table 2). The present review assessed the correlation between the occurrence and development of epilepsy and ferroptosis.

**TABLE 1:** The regulation of signaling pathways in ferroptosis.

Signaling pathway	References
**P53/SLC7A11/GPX4 pathway**	Wang et al., 2016
**NRF2/SLC7A11/GPX4 pathway**	Dong et al., 2020
**P62/Keap1/NRF2 pathway**	Sun et al., 2016; Fan et al., 2017
**Akt/NRF2/GPX4 pathway**	Gou et al., 2020
**GSK3β/NRF2/GPX4 pathway**	Wu et al., 2020
**NRF2/ARE pathway**	Dixon et al., 2012; Chandra et al., 2019
**System X_c_−/GSH/GPX4 pathway**	Bridges et al., 2012
**Iron metabolism pathway**	Liu J. et al., 2020
**Autophagy pathway**	Park and Chung, 2019
**FSP1-CoQ pathway**	Chen J. et al., 2020; Conrad and Proneth, 2020

**TABLE 2:** The inhibitors of signaling pathways in ferroptosis.

Influence factors	Inhibitors
**X_c_^–^**	Glutamate, erastin
**GPX4**	RSL3
**Lipid peroxidation**	Liproxstatin-1
**Fe^2+^**	Deferoxamine, bilirubin, ferrostatin-1
**ROS**	Antioxidant, lipid ROS scavengers
**Autophagy**	3-MA, chloroquine (CQ)

#### Reactive Oxygen Species (ROS)

Reactive oxygen species plays a major role in ferroptosis and epilepsy. In the cells, oxygen provides energy through oxidative phosphorylation in mitochondria (Wang H. et al., 2020). Oxygen (O_2_) is not only necessary for life but is involved in cell death. ROS is generated in many cellular processes, such as protein synthesis, mitochondrial metabolism, cellular respiration, metabolism of organic matter through a redox reaction, and tissue homeostasis (Mailloux et al., 2013; Qian et al., 2020). Also, ROS production is closely related to free radicals (Harris and DeNicola, 2020). In chemistry, free radicals exist in cells as atoms, molecules, or ions, such as OH^•^ and O^2–^, which have unpaired highly reactive valence electrons (Abramov et al., 2020). Excessive ROS causes cytotoxicity, while low concentrations can be released in the extracellular environment, protecting cells from bacterial damage via intracellular signaling molecules (Zhou Z. et al., 2020). Therefore, ROS concentration needs stringent control.

**FIGURE 2:**
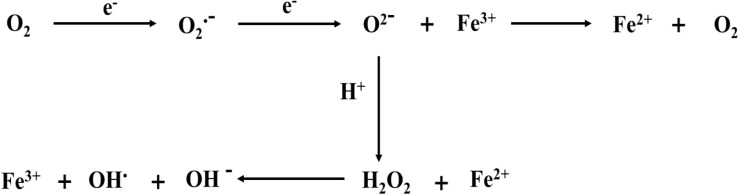
Iron ions are involved in the formation of free radicals. ROS originates from Fenton reaction, which is a process of producing OH^–^ and OH by the reaction of Fe^2+^ and H_2_O_2_. The Haber–Weiss cycle also shows that Fe^3+^ is reduced to Fe^2+^ through the reaction with O^2–^. and Fe^2+^ reacts with H_2_O_2_ to form OH, OH^–^, and Fe^3+^. The electron reaction with O_2_ at the mitochondrial electron transport chain produces O^2–^. Meanwhile, O^2–^ can bind hydrogen ions to produce H_2_O_2_.

The imbalance of intracellular oxidative stress appears to be a key factor in inducing ferroptosis. We also showed the correlation between iron ions and free radicals (Figure 2). During ferroptosis, ROS are produced from the Fenton reaction, which is a process of producing hydroxide (OH^–^) and hydroxyl (OH^•^) by the reaction between Fe^2+^ and H_2_O_2_ (Mittler, 2017). Moreover, The Haber–Weiss cycle showed that Fe^3+^ is reduced to Fe^2+^ through the reaction with superoxide (O^2–^), and Fe^2+^ reacts with H_2_O_2_ to form OH^•^, OH^–^, and Fe^3+^ (Capelletti et al., 2020). Thus, Fe^2+^ is conducive to the production of ROS and promotes lipid peroxidation, thus inducing ferroptosis. However, in hemorrhagic post-stroke epilepsy and PTE, large amounts of Fe^2+^ from red blood cells and hemoglobin are released into the brain, and these Fe^2+^ ions may be involved in inducing ferroptosis (Mori et al., 2004). In epileptic seizures, long-term seizures promote excessive ROS production and lead to the occurrence of oxidative stress, which is closely related to the generation of epileptic activities and the death of nerve cells (Freitas, 2009; Eastman et al., 2020). However, the rise of ROS leads to lipid peroxidation, leading to the occurrence of ferroptosis. Therefore, reasonable control of the ROS level reduces the occurrence of ferroptosis and may be conducive to delaying the occurrence and development of epilepsy.

#### Regulation of Ferroptosis by Cysteine-Glutathione (Cys-GSH) Redox Axis

Previous studies have shown that oxidative stress causes ferroptosis (Angelova et al., 2020). Moreover, oxidative stress is also considered as one of the pathogenesis of epilepsy (Shekh-Ahmad et al., 2019). Nuclear factor erythroid 2-related factor 2 (NRF2) regulates oxidative stress. However, related studies show that the activation of NRF2 alleviates the occurrence and development of AD by enhancing the antioxidant defense, improving mitochondrial function, inhibiting neuroinflammation, and inhibiting ferroptosis (Qu et al., 2020). The clearance of ROS in the human body mainly depends on GSH, which is mainly composed of small molecular peptides composed of glutamate (Glu), cysteine (Cys), and glycine (Gly) (Xiao and Meierhofer, 2019). GSH is also a substrate for GPX4 and can reduce the incidence of ferroptosis (Ursini and Maiorino, 2020). The deficiency of glutamate, cysteine, and glycine affects the expression level of GSH in cells. However, intracellular Cystine concentration is regulated by cystine-glutamate transporter system (X_c_^–^), which consists of two subunits (light chain xCT and heavy chain 4F2hc), on the cell membrane (Liu L. et al., 2020; Yamaguchi et al., 2020). xCT is the cystine/glutamate antiporter solute carrier family 7 member 11 (SLC7A11) that enhances cystine uptake and GSH biosynthesis, thereby inhibiting oxidative stress and iron death (Liu L. et al., 2020). Also, xCT is mainly responsible for cystine/glutamate exchange and highly specific to Cys and glutamate (Bridges et al., 2012). When the system X_c_^–^ is inhibited, the production of GSH is decreased and the level of ROS and lipid peroxidation is increased, thereby leading to the occurrence of ferroptosis (Capelletti et al., 2020).

**FIGURE 3:**
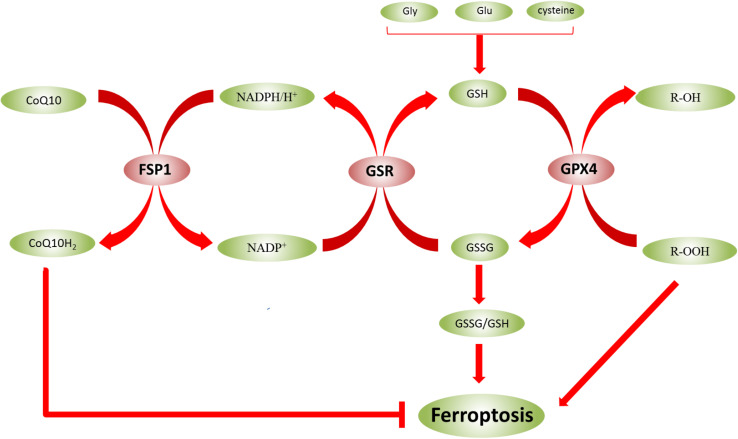
Glutathione (GSH) and GSSG can be converted to each other in the presence of enzymes. The ratio of GSSG/GSH indicates the degree of oxidative stress in cells. In biological reactions, GSH and GSSG can be converted into each other in the presence of enzymes. GSSG can be converted into GSH using NADPH/H^+^ as a cofactor under the catalysis of GSR, and NADP^+^ can also be converted to NADPH/H^+^. However, under the catalysis of FSP1, NADPH/H^+^ can be converted to NADP^+^, and CoQ10 is converted to CoQ10H_2_, which inhibits ferroptosis. GPX4 also converts GSH to GSSG. Moreover, lipid hydroperoxides (R-OOH) are converted to lipid alcohols (R-OH) by GPX4 using reduced GSH.

Glutathione exists in two forms—reduced (GSH) and oxidized (GSSG)—and the ratio of the two forms (GSSG/GSH) indicates the degree of oxidative stress in cells (Bjørklund et al., 2020). In biological reactions, GSH and GSSG can be converted into each other in the presence of enzymes (Figure 3). GSSG can be converted into GSH using NADPH/H^+^ as a cofactor under the catalysis of GSR. GSH is also a substrate for GPX4, which converts GSH to GSSG. Adjusting the GSSG/GSH ratio affects ROS levels (Bjørklund et al., 2020; Capelletti et al., 2020). However, in epilepsy, the GSH level was decreased, and the ROS level was increased. Therefore, the occurrence of ferroptosis might be inhibited by regulating the level of GSH to reduce the damage of nerve cells in the epileptic brain, which is beneficial to the treatment of epilepsy.

#### GPX4 and Ferroptosis

GPX4 is a protein that protects cell membranes against lipid peroxidation and converts GSH to GSSG. On the other hand, lipid hydroperoxides (R-OOH) are converted to lipid alcohols (R-OH) by GPX4 using reduced GSH as a cofactor (Figure 3) (Capelletti et al., 2020). This process prevents the formation and accumulation of iron-dependent lipid ROS. However, in ferroptosis, lipid ROS are more toxic than intracellular ROS. Also, lipids play a crucial role in the regulation of inflammation (Forcina and Dixon, 2019). Excessive cGPX4 detected in the cytoplasm and nucleus inhibits the production of leukotriene and prostaglandin. This phenomenon suggested that cGPX4 reduces fatty acid hydroperoxide that stimulates the activation of cyclooxygenase and lipoxygenase (Imai et al., 2017). Thus, GPX4 has a dual function in maintaining homeostasis in cells: as a guardian of antioxidant damage and a physiological regulator. However, the inhibition of GPX4 leads to ferroptosis via accumulation of lipid peroxides. Recent studies have shown the regulatory effects of RSL3 and erastin on GPX4 inactivation by directly binding GPX4 and indirectly reducing glutathione (Yang et al., 2014), respectively. Moreover, iron-dependent lipid peroxidation results in ferroptosis, which is inhibited by FSP1 and GPX4 (Bebber et al., 2020). In KA-induced epileptic models, the reduction in GPX4 expression, glutathione consumption, and the accumulation of lipid peroxides and iron were detected. Therefore, GPX4 may be a key molecule to prevent the death of hippocampal neurons caused by recurrent epileptic seizures and ultimately restore the cognitive function in TLE patients. In this review, we have the regulation of GPX4 in ferroptosis (Table 3).

**TABLE 3:** The regulation of GPX4 in ferroptosis.

Factors		Example	Mechanism	References
**Proteins**	**Nuclear factor**	**NRF2**	NRF2 regulates ferroptosis by NRF2/SLC7A11/GPX4 pathway, Akt/NRF2/GPX4 pathway, GSK3β/NRF2/GPX4 pathway, P62/Keap1/NRF2 pathway, NRF2/ARE pathway.	Sun et al., 2016; Fan et al., 2017; Dodson et al., 2019; Chen Y. et al., 2020; Dong et al., 2020; Gou et al., 2020; Wu et al., 2020; Yao et al., 2020; Zhou, 2020
	**Iron chaperone**	**PCBP1**	Iron chaperone PCBP1 can limit the toxicity of intracellular iron and prevent Ferroptosis caused by lipid peroxidation	Protchenko et al., 2020
	**Deacetylase**	**SIRT3**	SIRT3 deficiency is resistant to autophagy-dependent ferroptosis by inhibiting the AMPK/mTOR pathway and promoting GPX4 levels	Han et al., 2020
	**Histone demethylase**	**KDM3B**	Histone demethylase KDM3B protects against ferroptosis by upregulating SLC7A11.	Wang Y. et al., 2020
	**Metabolite**	**Bilirubin**	Bilirubin have the effect of chelating iron and can reduce ferroptosis of pancreatic islets, reduce oxidative stress, increase GPX4 expression, and up-regulate Nrf2/HO-1 expression.	Yao et al., 2020
	**Hormone**	**Melatonin**	Melatonin inhibits ferroptosis of neurons by Akt/Nrf2/GPX4 signaling pathway.	Gou et al., 2020
	**Mitochondrial electron transport chain**	**FSP1, CoQ**	CoQ, FSP1, and NAD(P)H act on the level of peroxyl radicals in membranes, thereby restraining lipid peroxidation. Iron-dependent lipid peroxidation leads to Ferroptosis, which is antagonized by GPX4 and FSP1	Bebber et al., 2020; Conrad and Proneth, 2020
		**CISDs**	CISDs exert an anti-ferroptotic function by suppressing free iron toxicity and lipid peroxidation.	Homma et al., 2020
**Vitamin**		**GCH1, BH**	GCH1and BH counteract ferroptosis through lipid remodeling and controlling endogenous production of the antioxidant BH, abundance of CoQ	Kraft et al., 2020
**Drug/chemical compound**		**PdPT**	PdPT, a broad-spectrum deubiquitinase inhibitor, can inhibit GPX4 proteasomal degradation, thereby inhibiting ferroptosis.	Yang J. et al., 2020
		**Irisin**	Irisin attenuated ischemia reperfusion (I/R)-induced AKI via upregulating GPX4.	Zhang J. et al., 2020
		**Gastrodin**	Gastrodin inhibits HO-induced ferroptosis through its antioxidative effect.	Jiang et al., 2020
		**Ginsenoside**	Ginsenoside mitigates 6-Hydroxydopamine-induced oxidative stress through upregulating GPX4	Lee et al., 2020
		**PACs**	PACs reduced the iron level in SCI, while the levels of GSH, GPX4, Nrf2, and HO-1 increased.	Zhou H. et al., 2020
		**CCA**	CCA inhibited ferroptosis by activation of system X_c_^–^/GPX4/Nrf2 and inhibition of NCOA4.	Zhou, 2020
**Internal environment**		**Hypertension**	Ferroptosis is closely related to hypertensive brain damage. Elevated blood pressure leads to iron overload which increases oxidative stress and lipid peroxidation in the brain	Yang L. et al., 2020
**External factors**		**Smoking**	Heavy smokers had significantly lower GSH levels and significantly higher lipid ROS and iron levels and GPX4 protein level decreased.	Ou et al., 2020
**Metal elements/non-metallic elements**		**ZnO, Co, Se**	ZnO, Co and Se triggered ferroptosis by elevation of ROS and lipid peroxidation, along with depletion of GSH and downregulation of GPX4.	Zheng et al., 2020

**FIGURE 4:**
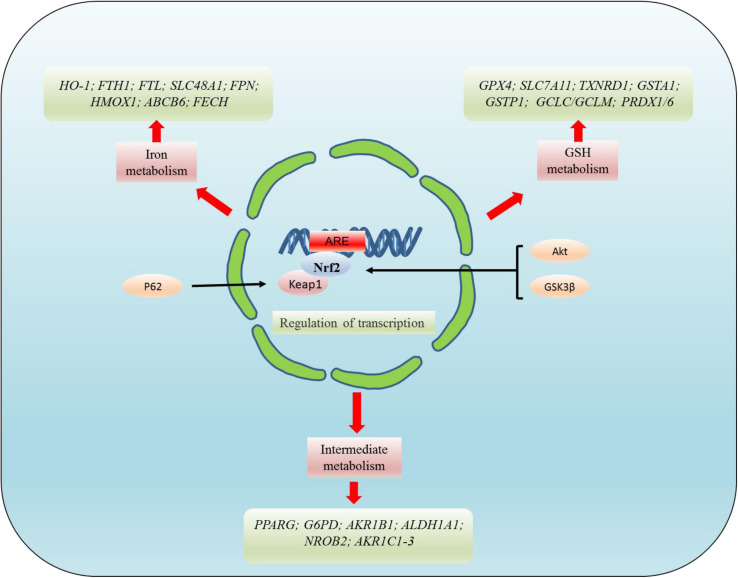
NRF2 regulates the targets related to ferroptosis at the transcriptional level. NRF2 mainly regulates the targets related to ferroptosis at the transcriptional level. These targets regulated by NRF2 can be divided into three broad classes: iron metabolism, intermediate metabolism, and GSH metabolism. However, NRF2 regulates a plethora of target genes involved in iron metabolism [*FTH1*, *FTL*, *SLC40A1*(FPN), *HMOX1*, *ABCB6*, *FECH*, *SLC48A1*, *BLVRA/B*, and HO-1], intermediate metabolism [*NROB2*(SHP), *PPARG*, *AKR1C1-3*, *G6PD*, *AKR1B1*, *ALDH1A1*], and GSH metabolism (*GPX4*, *SLC7A11*, *GCLC/GCLM*, *GSS*, *GSTA1*, *GSTP1*, *PRDX1/6*, and *TXNRD1*).

The transcription factor NRF2 plays a critical role in the regulation of GPX4. Current studies have shown that NRF2 deficiency enhances the susceptibility of PC12 cells to ferroptosis. However, the activation of NRF2 promotes GPX4 activity and iron storage capacity by increasing the level of GSH and the expression of ferritin heavy chain 1 (FTH1) (Liu Z. et al., 2020). Ferroptosis was also found to be closely related to the AKT/NRF2/GPX4 signaling pathway. However, melatonin inhibits the ferroptosis of neurons through the AKT/NRF2/GPX4 signaling pathway, thereby promoting the survival of hippocampus neurons and improving the hypoxic-ischemic brain injury (Gou et al., 2020). Meanwhile, GPX4 regulates erastin-induced ferroptosis via the GSK3/NRF2/GPX4 signaling pathway in breast cancer (Wu et al., 2020). Bilirubin chelates iron, while significantly increasing the expression of GPX4, upregulating NRF2/HO-1, and reducing ferroptosis in islet cells (Yao et al., 2020). In diabetics, cryptoxaneic acid (CCA) activates the Xc-/GPX4/NRF2 pathway and inhibits nuclear receptor coactivator 4 (NCOA4), thereby inhibiting ferroptosis (Zhou, 2020). Under physiological conditions, Keap1 stimulate NRF2 ubiquitination and proteasome degradation, thus reducing the level of NRF2 level. Conversely, NRF2 is activated in the process of oxidative stress, which activates the cell signaling pathway. This process can also be regulated by P62, which directly inhibits Keap1 and promotes the activation of NRF2. Relevant research has confirmed that ferroptosis is inhibited by the p62-Keap1-NRF2 pathway in hepatocellular carcinoma (HCC) (Sun et al., 2016). NRF2-Keap1 protein complex inhibits ferroptosis that regulates the antioxidant reaction, iron/heme metabolism, and carbohydrate and lipid metabolism (Dodson et al., 2019). The activation of NRF2-Keap1 signaling increases xCT (SLC7A11) and promotes glutamate secretion, while the inhibition of Keap1 enhances resistance to ferroptosis (Fan et al., 2017). NRF2 target genes are involved in preventing lipid peroxidation and ferroptosis. Furthermore, it was demonstrated that the activity of ferroptosis and lipid peroxide-related proteins are regulated by NRF2, a major regulator of antioxidant reactions. After oxidative stress, Keap1 degrades NRF2 and translocates it into the nucleus to initiate transcription of genes containing ARE, thereby regulating the transcription of ferroptosis-related proteins (Sun et al., 2016; Dodson et al., 2019). These targets are divided into three broad classes: iron metabolism, intermediate metabolism, and GSH metabolism (Figure 4). However, NRF2 regulates a plethora of target genes involved in iron metabolism [*FTH1*, *FTL*, *SLC40A1*(FPN), *HMOX1*, *ABCB6*, *FECH*, *SLC48A1*, *BLVRA/B*, and *HO-1*], intermediate metabolism [*NROB2*(SHP), *PPARG*, *AKR1C1-3*, *G6PD*, *AKR1B1*, and *ALDH1A1*], and GSH metabolism (*GPX4*, *SLC7A11*, *GCLC/GCLM*, *GSS*, *GSTA1*, *GSTP1*, *PRDX1/6*, and *TXNRD1*) (Dodson et al., 2019). In addition, the NRF2/HO-1 signaling pathway is also associated with ferroptosis-mediated ulcerative colitis (Chen Y. et al., 2020).

Another pathway that regulates GPX4 is the system X_c_^–^ -Gys-GSH redox axis. The constituent subunit of system X_c_^–^ includes light chain xCT (SLC7A11) and is regulated by many factors. Some studies demonstrated that histone demethylase KDM3B inhibits ferroptosis by upregulating SLC7A11 (Wang Y. et al., 2020). Moreover, acetylation of the lysine 98 residue is essential for p53-mediated ferroptosis (Wang et al., 2016). A relevant study has shown that the levels of H2Bub1 are reduced in erastin-induced ferroptosis, and the absence of H2Bub1 enhances the sensitivity of cells to ferroptosis. H2Bub1 also activates the expression of SLC7A11 via epigenetics. P53 negatively regulates H2Bub1 by promoting the nuclear translocation of the deubiquitinase enzyme USP7 (Wang et al., 2019). One target of p53^3KR^ [three lysines (K117R, K161R, and K162R) at p53 DNA binding sites mutate simultaneously] is *SLC7A11*, a gene encoding for the xCT (SCL7A11) of system Xc^–^. The binding downregulates the expression of SLC7A11, thereby affecting the activity of GPX4 (Jiang et al., 2015). In summary, the transcriptional inhibition of SCL7A11 leads to reduced antioxidant capacity, the accumulation of ROS, the lack of GPX4, and ferroptosis activation.

Current studies have found that some compounds, non-metallic elements, and metallic elements are involved in the regulation of GPX4, thereby regulating ferroptosis. GPX4 also plays an essential role in inhibiting autophagy-dependent ferroptosis induced by rapamycin and RSL3 (Liu Y. et al., 2020). It has been confirmed that GPX4 is a pivotal target for dihydroartemisinin (DHA)-activated ferroptosis in glioblastoma (Yi et al., 2020). Additionally, some metal ions are involved in the regulation of ferroptosis. Nano-Se inhibited Hcy-induced mitochondrial oxidative damage and apoptosis by inhibiting GPX1 and GPX4 in the vascular endothelial cells, thus effectively preventing vascular damage (Zheng et al., 2020). Furthermore, selenium (Se) is found in selenoprotein (GPX1, GPX3, GPX4, SELENOW, SELENOP, TXNRD2, and TXNRD3) in the form of selenocysteine (Sec) and is vital for the growth and development of vertebrates (Xu et al., 2020). GPX4 is essential to effectuate the transformation of Se. Zinc oxide nanoparticles (ZnO NPs)-induced cell death is involved in ferroptosis. Firstly, ferroptosis triggered by ZnO NPs is related to the increase in ROS, lipid peroxidation, deficiency of GSH, and the down-regulation of GPX4. Secondly, ZnO NPs can destroy intracellular iron homeostasis, also, p53 participates in ZnO NPs-induced ferroptosis. Finally, dissolved zinc ion contributes to ferroptosis (Zhang C. et al., 2020). In addition, Cobalt nanoparticles (Co NPs) and Cobalt (Co) salts elevate intracellular calcium, lipid peroxidation, GSH depletion, and inhibition of GPX4 expression. The Co-exposed cells were rescued by *N*-acetylcysteine (NAC), a part of liproxstatin-1 and an inhibitor of lipid peroxidation. The Co NPs-caused neuronal cell death might be related to ferroptosis mechanism (Gupta et al., 2020).

In addition to being regulated by the above pathways, ferroptosis is closely related to FSP1-CoQ10-NADPH pathway. It can also affect the sensitivity of ferroptosis. Moreover, NADPH is a critical reducing agent in cells and essential for balancing the lipid H_2_O_2_ levels. Some studies also suggested that the NADPH level is a biomarker of iron susceptibility in many cancer cell lines (Shimada et al., 2016). FSP1 is a protein that inhibits ferroptosis, compensates for GPX4 deficiency, and exerts a protective effect on ferroptosis. In addition, the inhibition of FSP1 on ferroptosis is mediated by coenzyme Q (CoQ10). FSP1 catalyzes the regeneration of CoQ10 through NADPH (Doll et al., 2019). CoQ10, as a part of the mitochondrial electron chain, not only participates in electron transport in the respiratory chain but also inhibits lipid peroxidation outside mitochondria by capturing free radical intermediates. Thus, the absence of CoQ10 might sensitize the cells to ferroptosis (Stockwell et al., 2020). Catalyzed by FSP1, NADPH/H^+^ is converted to NADP^+^ and CoQ10 is converted to CoQ10H_2_which inhibits ferroptosis (Figure 3). Recent studies have revealed a second major ferroptosis pathway, including FSP1, NADPH, and mitochondrial ubiquinone CoQ. Unlike the GPX4 pathway, this pathway affects the levels of peroxyl radicals on the membrane, thereby inhibiting lipid peroxidation (Conrad and Proneth, 2020). FSP1 catalyzes the production of CoQ10 through NADPH, and NADPH also inhibits ferroptosis through CoQ10 (Chen J. et al., 2020). Typically, FSP1 can compensate for the GPX4 deficiency in ferroptosis. GTP cyclohydrolase1 (GCH1) controls the production of the antioxidant tetrahydrobiopterin/dihydrobiopterin (BH/BH), the abundance of CoQ, and the peroxidation of unusual phospholipids via the GCH1-BH-phospholipid axis (Kraft et al., 2020). This study exhibits a unique mechanism of ferroptosis protection independent of the GPX4/GSH system. In addition, mitochondrial DNA stress triggers autophagy-dependent ferroptosis death (Li et al., 2020), and radiation-induced lipid peroxidation also triggers ferroptosis (Ye et al., 2020). These phenomena might be closely related to DNA damage, one of the causes of ferroptosis induced due to lipid peroxidation. Mitochondrial GPX4 also can inhibit the release of mitochondrial cytochrome C (cytC) by reducing H_2_O_2_ cardiolipin, thereby inhibiting apoptosis (Imai et al., 2017).

*Gpx4* is an essential gene for mouse embryonic development. The inactivation or silencing of *Gpx4* gene expression can result in the death of the embryo, possibly due to developmental defects in the brain (Forcina and Dixon, 2019). Gpx4 destruction also leads to the destruction of muscles, neurons, and other cells, suggesting that the survival of many cells requires GPX4 (Forcina and Dixon, 2019). Related studies have also shown that the genetic mutations in human *GPX4* are associated with sedaghatnia-type spondylometaphyseal chondrodysplasia, which causes bone, heart, and brain abnormalities and increased cell death (Smith et al., 2014). The majority of the studies on GPX4 involve ferroptosis. Strikingly, the synthesis and functional pathways of GPX4 are essential in regulating ferroptosis. GPX4 regulation is mainly related to system X_c_^–^ -Gys-GSH redox axis and NRF2 signaling pathways. The critical features of ferroptosis have been observed in the models of AD, epilepsy, stroke, and Parkinson’s disease (PD), including loss of glutathione, increased ROS, decreased GPX4, and lipid peroxidation. This suggests a possible link between these diseases and ferroptosis (Chen J. et al., 2020). It was also found that adult mouse hippocampal neuron loss and astrocyte proliferation occurred in the AD brain by inducing GPX4 deficiency (Yoo et al., 2012). In a hemorrhagic stroke model, injecting selenium into the brain boosted the expression of the antioxidant GPX4, thereby protecting the neurons and improving behavior (Alim et al., 2019). In addition, ferroptosis plays a crucial role in the acute and subacute phases of spinal cord injury. GPX4 inhibition induces iron prolapse in oligodendrocytes, while liproxstatin-1 effectively inhibits ferroptosis and may be a promising drug for the treatment of CNS diseases (Fan et al., 2021). In the subarachnoid hemorrhage (SAH) model, decreased GPX4 may play a major role in early brain injury after SAH. However, GPX4 overexpression significantly reduces lipid peroxidation and cell death and exerts a neuroprotective effect on SAH *in vitro* and *in vivo* (Gao et al., 2020). In the epileptic models, loss of hippocampal neurons may be due to reduced GPX4 expression, glutathione consumption, lipid peroxides, and iron accumulation (Qing et al., 2019). Genetic engineering of mice, where Sec was replaced with Cys at the *Gpx4* active site, could lead to fatal postnatal epileptic seizures owing to the loss of parvalbumin-positive interneuron (Friedmann Angeli and Conrad, 2018). Some studies have also found that animals with *Trsp* and *Gpx4* knockout exhibit significant neurophenotypes, including hyperexcitability, spontaneous epilepsy, and ataxia (Fan et al., 2021). Therefore, GPX4 may be a key molecule to prevent the death of hippocampal neurons caused by recurrent epileptic seizures and ultimately restore the cognitive function of TLE patients. Taken together, the pharmacological inhibition of GPX4 leads to the accumulation of lipid peroxides, subsequently leading to ferroptosis. Ferroptosis may contribute to the elimination of cancer cells resistant to treatment in order to enhance the effect of chemotherapy or radiotherapy. On the other hand, GPX4 may have neuroprotective functions, and the regulation of GPX4 might be a new strategy for the treatment of neurodegenerative diseases including epilepsy. However, whether GPX4 can be a putative target for reducing neuronal injury in epileptic brain, especially in PTE, is yet to be verified in animal models and cell epilepsy models.

#### Iron Metabolism and Ferroptosis

Iron homeostasis is controlled in cells and systems, such as to provide the iron needed for cells and tissues, thereby avoiding iron overload and iron-related toxicity (Piperno et al., 2020). In the human body, iron is found in both Fe^2+^ and Fe^3+^ forms, which is used to synthesize metalloproteins to participate in the human body (Daher et al., 2017). Heme iron and non-heme iron from food is absorbed in the gut in different ways.

Heme and non-heme iron from dietary sources exhibit different absorption abilities in the gut. HCP-1 internalizes iron in the heme form but not the non-heme form. However, heme is rapidly catabolized by HO-1 to release the iron (Daher et al., 2017). Fe^3+^ can be reduced into the Fe^2+^ form by DCYTB, and Fe^2+^ is imported by the apical DMT1 (Schlottmann et al., 2017). In the basolateral membrane of intestinal epithelial cells, ferritin (FPN) is the main iron exporter, transporting Fe^2+^. At the basolateral side, Fe^2+^ is oxidized to Fe^3+^ by ceruloplasmin. Fe^3+^ binds to plasma transferrin (TF) to transport iron to all organs, including the brain (Daher et al., 2017). Transferrin delivers iron into the cells by binding to transferrin receptor-1 (TFR1). After Fe^3+^ enters the cell via membrane protein TFR1, Fe^3+^ is released from TF. Fe^3+^ is then reduced to Fe^2+^ by the metalloreductase (STEAP3) in endosomes (DeGregorio-Rocasolano et al., 2018). Iron can be stored in ferritin or transported by FPN and is released from endosomes into the LIP of the cytoplasm by DMT1, thereby keeping unstable iron pools at low levels and avoiding cytotoxicity (Ohgami et al., 2005; Martin-Sanchez et al., 2020). As shown in Figure 2, when an intracellular iron overload occurs, excess iron promotes lipid peroxidation through ROS production and Fenton reaction, thus causing ferroptosis (Bogdan et al., 2016). Thus, a close correlation has been established between iron metabolism genes and iron toxicity sensitivity. For example, the silencing of *TFRC* gene encoding TRF1 inhibits erastin-induced ferroptosis and prevents LIP accumulation, while HO-1 promotes ferroptosis by supplementing iron (Kwon et al., 2015). Moreover, the silencing of IREB2, as the iron metabolism master regulator, also decreases the sensitivity of cells to ferroptosis (Dixon et al., 2012). IREB2 silencing alters many genes in iron metabolism: *TRFC*, *FTH1*, and *FTL* (Capelletti et al., 2020). Some studies have confirmed that HSBP1 reduces iron levels by inhibiting TFR1 expression. Phosphorylated HSPB1, is a negative regulator of ferroptosis that reduces iron uptake and lipid peroxidation, thereby decreasing eracetin-mediated ferroptosis (Sun et al., 2015). Thus, iron metabolism in cells, including iron absorption, output, utilization, and storage, is closely related to ferroptosis (Liu J. et al., 2020). Concurrently, monitoring the unstable iron ions in mitochondria, lysosomes, and endoplasmic reticulum during ferroptosis may be a good strategy to study the diseases, such as epilepsy, AD, and stroke, of the CNS. In conclusion, iron metabolism is closely related to the occurrence of ferroptosis.

#### Autophagy-Dependent Ferroptosis

According to current studies, excessive autophagy promotes ferroptosis through iron accumulation or lipid peroxidation. The activation of ferroptosis requires transferrin receptors and transferrin to transport iron outside the cells. However, autophagy can also regulate the sensitivity of cells to ferroptosis by affecting iron metabolism (Gao et al., 2015; Hou et al., 2016). In addition, autophagy results in iron-dependent ferroptosis by regulating the induction of TFR1 and degradation of ferritin (Park and Chung, 2019). Some studies demonstrated that nuclear receptor coactivator 4 (NCOA4)-induced ferritinophagy is closely related to neurodegeneration (Quiles Del Rey and Mancias, 2019). Ferritinophagy, a ferritin degradation pathway, depends on NCOA4 (Lin et al., 2020), a selective cargo receptor for the autophagic turnover of ferritin by lysosomes (Mancias et al., 2014). It has been found that ferritinophagy is involved in FC-induced ferroptosis in the cells with elevated NCOA4 (Lin et al., 2020). The downregulation of NCOA4 or ATGs (ATG5, ATG7, and ATG13) decreases ferroptosis by inhibiting of ferritin degradation, iron accumulation, and lipid peroxidation (Hou et al., 2016; Xie et al., 2016). Ferritin degradation occurs mainly in lysosomes and leads to iron release and oxidative damage. Previous studies have shown that ferritinophagy is a process of ferritin degradation by autophagy that promotes ferroptosis (Hou et al., 2016; Xie et al., 2016). In addition, ferritinophagy is crucial for the inhibition of hepatic fibrosis mediated by ferroptosis (Zhang Z. et al., 2018; Kong et al., 2019). Dihydroartemisinin (DHA) induces ferritinophagy by AMPK/mTOR/p70S6k signaling pathway. However, the activation of ferritinophagy promoted the degradation of ferritin, increased the labile iron pool, and eventually induced ferroptosis (Du et al., 2019).

In addition to ferritinophagy, lipophagy also regulates ferroptosis. Lipophagy is also a form of selective autophagy that leads to the autophagic degradation of intracellular lipid droplets (LDs) (Liu and Czaja, 2013). Some studies have shown that lipophagy promotes ferroptosis induced by RSL3 in hepatocyte cells. Importantly, *in vitro* and *in vivo*, both the blocking of ATG5- and RAB7A-dependent lipid degradation and the enhancement of TPD52-dependent lipid storage prevents RSL3-induced lipid peroxidation and subsequent ferroptosis (Bai et al., 2019).

In addition, ferroptosis is also associated with a number of autophagy-related pathways. In myocardial cells after myocardial infarction (MI), miR-30d inhibited autophagy by binding to ATG5 and subsequently enhanced ferroptosis (Tang et al., 2020). ROS-autophagy-lysosome pathway promotes the ATPR-induced differentiation of acute myeloid leukemia (AML) through targeting ferroptosis (Du et al., 2020). Furthermore, arsenic can also result in pancreatic dysfunction and ferroptosis via the mitochondrial ROS-autophagy-lysosomal pathway (Wei et al., 2020). Accumulating evidence has shown that the interaction between mTOR and GPX4-related signaling pathways regulate the role of autophagy-dependent ferroptosis in human pancreatic cancer cells (Liu Y. et al., 2020). Additional studies have shown that the upregulation of sirtuin3 (SIRT3) promotes the AMPK-mTOR pathway and reduces GPX4 level to enhance autophagy activation, leading to ferroptosis in trophoblast cells (Han et al., 2020). Mitochondrial DNA stress triggers autophagy-dependent ferroptosis via STING1/TMEM173-mediated DNA sensing pathway (Li et al., 2020). Through the release and uptake of oncogenic KRAS protein, tumor-associated polarization of macrophage can also be driven by autophagy-dependent ferroptosis (Dai et al., 2020). The inhibition of RNA binding protein ZFP36/TTP for ferroptosis is closely related to regulating autophagy signaling pathway in hepatic stellate cells (Zhang Z. et al., 2020). The selective degradation of ARNTL, a core circadian clock protein, by autophagy is the key to ferroptosis. ARNTL also inhibits the transcription of *EGLN2* to repress ferroptosis, leading to the activation of HIF1A, a prosurvival transcription factor (Yang et al., 2019). Other studies have shown that chaperone (HSP90)-mediated autophagy (CMA) is involved in the execution of ferroptosis (Wu et al., 2019). In addition, it is demonstrated that the inhibition of autophagy-mediated HDAC enhances HMGB1 acetylation, consequently releasing HMGB1 in ferroptosis. Thus, HMGB1 is a key regulator of autophagy (Wen et al., 2019).

In conclusion, autophagy induces ferroptosis and is also related to the occurrence and development of epilepsy. Therefore, the study of autophagy-dependent ferroptosis is beneficial to control nerve damage in the epileptic brain, thereby reducing epileptic seizures.

### Ferroptosis in Epilepsy

Previous studies have shown that early persistent dysregulation of iron metabolism and activation of antioxidant signals are pathological features of focal cortical dysplasia type IIb (FCD IIb) and tuberous sclerosis complex (TSC) (Zimmer et al., 2020). High levels of ROS and oxidative stress are detected in nerve cells after an epileptic seizure. Simultaneously, oxidative stress further aggravates nerve damage (Freitas, 2009; Eastman et al., 2020), i.e., in acute brain damage, such as stroke and epilepsy, the brain tissue is particularly vulnerable to oxidative stress due to consumption of a large amount of oxygen and produces a large number of free radicals as compared to other tissues. However, in hemorrhagic post-stroke epilepsy and PTE, bleeding in the villi blood vessels in the brain causes hemolysis and hemoglobin deposition in the neocortex. Hemoglobin and iron produced by the transfer are deposited in the brains of individuals with PTE. Iron compounds form reactive free radical oxidants (Willmore and Ueda, 2009). As observed in epileptic patients, microinjections of trivalent iron ions into rodent brains lead to chronic recurrent episodes and release of glutamate into nerve fibers (Willmore and Ueda, 2009). However, in hemorrhagic post-stroke epilepsy and PTE, large amounts of Fe^2+^ are released from red blood cells and hemoglobin into the brain, and these Fe^2+^ ions might ferroptosis. These studies suggested that ferroptosis occurs in hemorrhagic post-stroke epilepsy and PTE, and Fe^2+^ plays a major role in regulating ferroptosis in epilepsy.

Some studies have shown that high level of extracellular glutamate is an inhibitor of system X_c_^–^ (Fricker et al., 2018). However, a high level of extracellular glutamate occurs in the brain during epileptic seizures and is one of the causes of recurrent seizures. In addition, the high level of extracellular glutamate promotes epileptic seizures and status epilepticus (SE) in TLE (Albrecht and Zielińska, 2017). The inhibition of system X_c_^–^ determines the decline in glutathione levels and the onset of ferroptosis (Liu L. et al., 2020). The concentration of extracellular glutamate might be an effective inhibition strategy to prevent Cys uptake and induce ferroptosis. Furthermore, GPX4 expression was decreased in epilepsy but was increased in nerve injury after the use of iron death inhibitors (Mao et al., 2019). Targeting ferroptosis is also regarded as a novel therapeutic strategy for epilepsy associated with mitochondrial dysfunction (Kahn-Kirby et al., 2019). Reportedly, the combination of iron supplements reduces serum carbamazepine concentration (Perucca, 2018). These studies suggested that ferroptosis occurs in epilepsy, and glutamate may play a role in regulating this ferroptosis in epilepsy.

Importantly, ferroptosis has been confirmed in epilepsy. Iron chelators, including Deferoxamine (DFO) and antioxidant molecules, such as errostatins, liproxstatin-1, and vitamin E, are also inhibited pharmacologically. In conclusion, ferroptosis plays a major role in the occurrence and development of epilepsy.

## Treatment of Epilepsy by the Regulation of Iron Metabolism and Ferroptosis

Current studies have shown that reducing nerve damage in epilepsy by targeting ferroptosis and iron metabolism delays the onset and progression of epilepsy. However, the role of iron death and the regulation of iron metabolic pathways in the treatment of epilepsy is yet to be elucidated. According to the above mechanisms of ferroptosis and iron metabolism regulation, some proteins, drugs, and external and internal factors affect ferroptosis and iron metabolism in epilepsy.

CDGSH iron-sulfur domain protein (CISDs) resist ferroptosis by inhibiting free iron toxicity and lipid peroxidation in the presence of cysteine (Homma et al., 2020). NRF2 prevents acute lung injury (ALI) by regulating SLC7A11 and HO-1 that inhibits the effect of pulmonary ferritin (Dong et al., 2020). Deubiquitinase inhibitors, such as palladium pyridine thione complex (PdPT), have been found to inhibit GPX4 proteasomal degradation, thereby inhibiting ferroptosis. This phenomenon revealed a new mechanism of GPX4 posttranslational modification in the ferroptosis and a possible strategy of the inhibition of ferroptosis by protein deubiquitination inhibition (Yang J. et al., 2020). The upregulation of GPX4 leads to an irisin-mediated decrease in acute kidney injury (AKI) induced by ischemia-reperfusion (I/R). However, the protective effect of irisin was disrupted by GPX4 inhibitor RSL3 (Zhang J. et al., 2020). Ferroptosis is closely related to hypertensive brain injury. In the brain, elevated blood pressure results in iron overload, which increases oxidative stress and lipid peroxidation, eventually leading to brain injury (Yang L. et al., 2020). Proanthocyanidin (PAC) significantly reduces the iron content in patients with spinal cord injury (SCI), while the levels of GPX4, GSH, NRF2, and HO-1 are increased. This suggested that PAC may be a novel drug that inhibits ferroptosis (Zhou H. et al., 2020). In heavy smokers, GSH levels were significantly lower and lipid ROS and iron levels were significantly higher than the non-smoking group. In addition, lipid ROS and iron level increased, GSH level decreased, and GPX4 protein level decreased after cigarette smoke condensate (CSC) treatment (Ou et al., 2020). Iron companion Poly rC binding protein 1 (PCBP1) prevents iron sag caused by lipid peroxidation, thus reducing intracellular iron toxicity (Protchenko et al., 2020). Dexmedetomidine inhibits sepsis-induced myocardial ferroptosis by reducing iron concentration and upregulating GPX4, thereby decreasing septic heart injury (Wang C. et al., 2020). Gastrodin inhibits HO-induced ferroptosis through the antioxidative effect in rat glioma cell line C6 (Jiang et al., 2020). Ginsenoside upregulates GPX4 to mitigate oxidative stress induced by 6-hydroxydopamine (Lee et al., 2020). These proteins, drugs, and external and internal factors may affect ferroptosis and iron metabolism in epilepsy but have not yet been explored.

Some studies have shown that valproic acid (VPA) treatment contributes to iron metabolism in epilepsy, leading to the formation of non-transferrin-bound iron (NTBI) and an increase in oxidative stress. However, other iron status parameters (serum iron, ferritin, and transferrin saturation) were normal in epileptic patients (Ounjaijean et al., 2011). Dehydroepiandrosterone (DHEA) treatment exerts antiepileptic effects on iron-induced local epileptiform brain activity in rats. It alleviates cognitive deficits associated with epileptic activity while inhibits lipid peroxidation, protein oxidation, and Na(+)/K(+)-ATPase (sodium pump) activity in epilepsy (Monika et al., 2010). Recent studies have also reported that DHEA reduces oxidative stress and apoptosis of iron-induced epilepsy via activation of the NRF2/ARE signaling pathway (Chandra et al., 2019). Similarly, PTE appears to be caused by a series of events, such as bleeding, hemolysis, the release of iron or heme compounds, free radical formation, lipid peroxidation, and cell death, the free radical scavenging agents and antioxidants include diphosphate of vitamin E and C, melatonin, vanillin (Pagni and Zenga, 2005). The results showed that the oxidative damage of nerve cells in iron-induced epilepsy was attenuated by α-phenyl tert-*N*-butyl nitroketone (PBN) treatment, a nitroketone free radical scavenger (Carolina et al., 2003). Previous studies have shown that α-tocopherol-L-ascorbic acid-2-*O*-phosphodiester potassium salt (EPC-K1), a OH. scavenger, protects the oxidation of nerve cell membranes and prevents epileptic discharges caused by iron ions (Nihei et al., 2002). Intriguingly, 30 min before FeCl_3_ injection, intraperitoneal injection of adenosine dichloride (Cl-Ado) or adenosine (Ado) inhibited or delayed the occurrence of FeCl_3_-induced epileptic discharge. Cl-ado and Ado inhibit the occurrence of epileptic discharge by scavenging hydroxyl radicals and their anticonvulsant effects (Yokoi et al., 1995). Fisetin reduces lipid peroxides and retains Na (+), K (+)-ATPase activities in PTE, and executes an antiepileptic role in iron-induced epileptic rat models by inhibiting oxidative stress and reduces the cognitive dysfunction related to epileptic seizures (Jharana et al., 2017). Epileptic brain fluorescent imaging revealed that apigenin relieves myeloperoxidase-mediated oxidative stress and inhibits ferroptosis (Shao et al., 2020).

An increasing number of neurological diseases, including epilepsy, are involved in the activation of ferroptosis. Therefore, targeted ferroptosis is a novel strategy for the treatment of epilepsy. Studies have shown that inhibition of ferroptosis improves cognitive dysfunction in rats with temporal lobe epilepsy induced by KA. Ferrostatin-1, a specific iron death inhibitor, was treated to prevent the development of hypertrophy in the hippocampus of KA-treated rats. This phenomenon could be attributed to the decreasing of GPX4 expression, GSH depletion and lipid peroxides, and iron accumulation. Ferrostatin-1 was also shown to prevent the loss of KA-induced hippocampal neurons and restore cognitive function in TLE rats (Qing et al., 2019). Simultaneously, the level of local transferrin decreased after treatment with iron-chelating agent deferoxamine (DFO). DFO removes iron and controls epilepsy, indicating its potential therapeutic value in patients with refractory epilepsy after hemorrhagic stroke (Xiang et al., 2017). In addition, the inhibition of 5-lipoxygenase inhibitor (Zileuton) for glutamate-and erastin-induced ferroptosis in HT22 hippocampus cells is similar to ferrostatin-1. According to the *in vivo* and *in vitro* studies, the lipoxygenase inhibitor has exhibited a neuroprotective activity (Liu et al., 2015; Conrad et al., 2016).

Taken together, studies have shown that targeting iron death and iron metabolism is beneficial to reduce nerve death and seizures in epilepsy (Table 4). Currently, there is no systematic and in-depth study on the role of iron death and iron metabolism pathways in epilepsy. Thus, the role of these iron death and iron metabolism pathways in epilepsy need further investigation.

**TABLE 4:** Study on epilepsy treatment about iron metabolism and ferroptosis.

Drug	Mechanism	References
**Valproic acid (VPA)**	VPA contributes to iron metabolism in epilepsy, leading to the formation of non-transferrin-bound iron (NTBI) and an increase in oxidative stress	Ounjaijean et al., 2011
**Carbamazepine**	The combination of iron supplements can reduce serum carbamazepine concentration	Du et al., 2020
**Dehydroepiandrosterone (DHEA)**	DHEA had antiepileptic effects and alleviated cognitive deficits associated with epileptic activity. DHEA can inhibit lipid peroxidation, protein oxidation, and Na^+^, K^+^-ATpase activity in epilepsy. DHEA can reduce oxidative stress and apoptosis of epilepsy iron-induced by activating the NRF2/ARE signaling pathway.	Chandra et al., 2019
**Vitamin E and C, melatonin, vanillin**	It has antioxidant function, scavenging free radicals and reducing ROS production.	Pagni and Zenga, 2005
**α-phenyltert-*N*-butyl nitroketone (PBN)**	The oxidative damage of nerve cells in iron-induced epilepsy was attenuated by PBN	Carolina et al., 2003
**EPC-K1**	EPC-K1, as hydroxyl radical scavenger, protects the oxidation of nerve cell membranes and prevents epileptic discharges caused by iron ions	Nihei et al., 2002
**Adenosine dichloride (Cl-Ado) or adenosine (Ado)**	Cl-Ado or Ado inhibited or delayed the occurrence of FeCl_3_-induced epileptic discharge by scavenging hydroxyl radicals and their anticonvulsant effects	Yokoi et al., 1995
**Fisetin**	Fisetin can reduce lipid peroxides and retain Na^+^, K^+^-ATpase activities in post-traumatic epilepsy. Meanwhile, fisetin can play an anti-epileptic role in iron-induced epileptic rat models by inhibiting oxidative stress.	Jharana et al., 2017
**Ferrostatin-1**	Ferrostatin-1 can prevent the development and development of hypertrophy in the hippocampus of KA-treated rats by decreasing GPX4 expression, GSH depletion and lipid peroxides and iron accumulation. Ferrostatin-1 was also found to prevent the loss of KA-induced hippocampal neuron and restore cognitive function in TLE rats	Qing et al., 2019
**Deferoxamine (DFO)**	Transferrin decreased after iron chelating agent deferoxamine (DFO) treatment. DFO can effectively remove iron and control epilepsy after hemorrhagic stroke	Xiang et al., 2017
**Zileuton**	The inhibition of 5-lipoxygenase inhibitor Zileuton on glutamate-and Erastin-induced iron poisoning in HT22 hippocampus cells is similar to statin-1, and the lipoxygenase inhibitor has been shown to have neuroprotective activity *in vivo* and *in vitro*.	Liu et al., 2015; Conrad et al., 2016

## Conclusion

In this review, we elucidated the regulatory mechanism of ferroptosis, iron metabolism, and ferroptosis in epilepsy, and the effect of targeted ferroptosis and iron metabolism on epilepsy treatment. Increasing evidence demonstrates that epilepsy is closely related to ferroptosis and iron metabolism. In PTE or post-hemorrhagic stroke epilepsy, hemoglobin iron ions are released in abundance. Subcortical injections of iron can also induce seizures. Currently, there are many ways to regulate ferroptosis, but the regulatory mechanism of ferroptosis in epilepsy has not yet been understood. Anti-ferroptosis for epilepsy treatment is still at the animal level and has not been tested in clinical trials. Thus, targeting iron metabolism and ferroptosis may be a novel method to slow the progression of epilepsy. This type of treatment is yet to be discovered and explored. Nonetheless, ferroptosis is a newly identified and critical mechanism of cell death, especially in CNS disease. Anti-ferroptosis mechanisms and ferroptosis inhibitors play an essential role in preventing epilepsy, especially PTE or post-hemorrhagic stroke epilepsy.

## Author Contributions

SC participated in experimental studies. SC and YC searched and sorted out the references and participated in drafting the manuscript. SC and XK were involved in the literature search. QL and DZ coordinated and supervised the work, provided research direction, designed research plans, and modified the final drafts. All authors have carefully read and confirmed the final manuscript.

## Conflict of Interest

The authors declare that the research was conducted in the absence of any commercial or financial relationships that could be construed as a potential conflict of interest.

## Abbreviations

AD: Alzheimer’s disease
AEDs: antiepileptic drugs
ARE: antioxidant response elements
CNS: central nervous system
Cys: cysteine
SLC7A11: cystine/glutamate antiporter solute carrier family 7 member 11
CoQ10: coenzyme Q10
DFO: deferoxamine
DCYTB: duodenal cytochrome B reductase
FSP1: ferroptosis suppressor protein 1
FeCl_3_: ferric chloride
DMT1: divalent metal transporter 1
GSH: glutathione
GSR: glutathione reductase
GPX4: glutathione peroxidase 4
Glu: glutamate
Gly: glycine
HCP-1: heme carrier protein-1
HO-1: heme oxygenase 1
OH^•^: hydroxyl radical
H_2_O_2_: hydrogen peroxide
HS: hippocampus sclerosis
IREB2: iron-responsive element-binding protein 2
KA: kainic acid
Keap1: Kelch-like ECH-associated protein 1
MTLE: mesial temporal lobe epilepsy
Nrf2: nuclear factor erythroid 2 related factor 2
NCOA4: nuclear receptor co-activator 4
PD: Parkinson’s disease
PTE: post-traumatic epilepsy
ROS: reactive oxygen species
RNS: reactive nitrogen species
TBI: traumatic brain injury
O^2–^: superoxide anion
STEAP3: six-transmembrane epithelial antigen of prostate 3
SIRT3: Sirtuin3
SAH: subarachnoid hemorrhage
TLE: temporal lobe epilepsy
TF: transferrin
TFR1: transferrin receptor-1.
